# LAT1 and ASCT2 Related microRNAs as Potential New Therapeutic Agents against Colorectal Cancer Progression

**DOI:** 10.3390/biomedicines9020195

**Published:** 2021-02-16

**Authors:** Francisca Dias, Cristina Almeida, Ana Luísa Teixeira, Mariana Morais, Rui Medeiros

**Affiliations:** 1 Molecular Oncology and Viral Pathology Group, IPO-Porto Research Center (CI-IPOP), Portuguese Oncology Institute of Porto (IPO-Porto), Research Center—LAB2, E Bdg 1st Floor, Rua Dr António Bernardino de Almeida, 4200-072 Porto, Portugal; Francisca.Carvalho.Dias@ipoporto.min-saude.pt (F.D.); cristinalmeida11@hotmail.com (C.A.); mariana.gomes.morais@ipoporto.min-saude.pt (M.M.); ruimedei@ipoporto.min-saude.pt (R.M.); 2 Research Department of the Portuguese League against Cancer Regional Nucleus of the North (LPCC-NRN), Estrada da Circunvalação 6657, 4200-177 Porto, Portugal; 3 Institute of Biomedical Sciences Abel Salazar, University of Porto (ICBAS-UP), Rua Jorge Viterbo Ferreira 228, 4050-513 Porto, Portugal; 4 Faculty of Medicine, University of Porto (FMUP), Alameda Prof. Hernâni Monteiro, 4200-319 Porto, Portugal; 5 Biomedical Research Center (CEBIMED), Faculty of Health Sciences of Fernando Pessoa University (UFP), Praça 9 de Abril 349, 4249-004 Porto, Portugal

**Keywords:** colorectal cancer, amino acid transporters, LAT1, ASCT2, miRNAs

## Abstract

The development and progression of colorectal cancer (CRC) have been associated with genetic and epigenetic alterations and more recently with changes in cell metabolism. Amino acid transporters are key players in tumor development, and it is described that tumor cells upregulate some AA transporters in order to support the increased amino acid (AA) intake to sustain the tumor additional needs for tumor growth and proliferation through the activation of several signaling pathways. LAT1 and ASCT2 are two AA transporters involved in the regulation of the mTOR pathway that has been reported as upregulated in CRC. Some attempts have been made in order to develop therapeutic approaches to target these AA transporters, however none have reached the clinical setting so far. MiRNA-based therapies have been gaining increasing attention from pharmaceutical companies and now several miRNA-based drugs are currently in clinical trials with promising results. In this review we combine a bioinformatic approach with a literature review in order to identify a miRNA profile with the potential to target both LAT1 and ASCT2 with potential to be used as a therapeutic approach against CRC.

## 1. Introduction

Colorectal cancer (CRC) is one of the most common cancers worldwide, with 1,849,518 new cases in 2018, being the third most common cancer [1]. Currently, CRC accounts for approximately 10% of all diagnosed cancers and it is the world’s second most deadly cancer [2]. CRC is the second most common neoplasia diagnosed in women, and the third in men, being the incidence and mortality approximately 25% lower in woman [2]. CRC development can be modulated by several factors, being the high alcohol consumption, overweigh, physical inactivity, tobacco smoking, diabetes mellitus, age, personal or family history of CRC well established risk factors [3,4]. Although the mortality rates have declined due to the improvement in diagnosis and treatment, CRC still represents one of the most lethal cancer types [3]. Furthermore, metastasis is also found in, approximately, 15–25% of CRC cases at the diagnosis, and increase to 50% during the course of the disease [2,5]. The advances in the pathophysiological and molecular CRC knowledge allowed the increase of the treatment options, but these new therapeutic approaches were proven to be more effective in patients with non-metastatic disease [2]. Thus, it is imperative to clarify the mechanisms involved in disease progression, aggressiveness and metastasis formation in order to improve the patients’ follow up and to identify new therapeutic approaches.

Recently, the literature showed that amino acid (AA) transporters, such as solute carrier transports (SLCs), are important players in tumor development, since tumor cells have an increased demand for AA to sustain their excessive proliferation rate [6]. In fact, it is described that tumor cells upregulate some AA transporters in order to support the increased AA demand and intake [7]. Moreover, SLCs are able to regulate the PI3K/Akt/mTORC1 signaling pathway, which is central in the regulation of CRC proliferation and aggressiveness and is also involved in metabolic reprograming [8,9]. In fact, there are some SLC dysfunctions associated with CRC, such as L-type amino acid transporter 1 (LAT1) and alanine-serine-cysteine transporter 2 (ASCT2) upregulation, that may have an impact on disease aggressiveness [9,10,11]. Given the growing evidence and interest in the impact of cancer metabolism in disease aggressiveness, it is imperative to further understand the regulatory mechanisms responsible for of LAT1 and ASCT2 modulation in CRC and study the potential of their inhibition as a therapeutic approach. Since these two AA transporters are frequently overexpressed in CRC cells, they have potential as drug targets because their inhibition or blockade could lead to cell cycle arrest and apoptosis [10,12,13,14].

### 1.1. Amino Acid Transporters Deregulation in CRC: The Impact of LAT1 and ASCT2

It has been nearly a century since the discovery that normal and tumor cells differ in energy metabolism, with tumor cells presenting a higher need of nutrients, being the AA bioavailability crucial to support cell proliferation and growth [15]. Amino acids can be classified into three groups: (1) essential AA (EAA), if the organism is not able to synthesize them and needs to acquire them from the diet; (2) non-essential AA, if they are synthesized in sufficient quantities by the organism or (3) conditional AA, if are usually nonessential, except in times of illness, trauma or stress were they become conditionally essential [16,17].

In addition to their need in protein synthesis, several amino acids have other roles in supporting cancer development. One example is glutamine, the most abundant AA that participates in energy production, redox homeostasis, macromolecular synthesis and cell signaling [18]. In fact, the commitment of glutamine in the these cell processes makes this AA conditionally essential in conditions characterized by a high proliferation rate, such as cancer, in which endogenous glutamine synthesis is not sufficient to satisfy the cell need [17].

Since AAs are hydrophilic, they need selective transport proteins in order to cross the plasma membrane of the cells. There are approximately two-dozen amino acid transporters in humans, and cancer cells must regulate one or more of these transporters to satisfy their nutrient demand [6]. LAT1 (SLC7A5) is a transmembrane transporter involved in the import of large and neutral AA such as leucine and phenylalanine, in exchange for intracellular AA, such as glutamine [10,13,19]. According to various studies, LAT1 is highly upregulated in multiple human cancers, including gastrointestinal cancers [10,19,20,21]. In fact, Hayase and coworkers found a higher expression of LAT1 in 72.4% of CRC cases when compared to colonic adenoma cases, concluding that LAT1 could be a marker for malignant lesions [10]. Furthermore, Zhang and colleagues also found an association of higher LAT1 expression levels to poorer outcomes and shorter survival in several types of cancer, including CRC [14]. The higher LAT1 expression in cancer cells shows the importance of this AA transporter in the maintenance of AA nutrition in cancer cells [6]. Studies conducted by Elorza and coworkers show that the upregulation of LAT1 is involved in the increase of mTORC1 activity through HIF2α activation, showing a relationship between the hypoxic microenvironment, HIF2α and LAT1 [22]. Furthermore, LAT1 mediates leucine uptake with high affinity, which is a key AA activator of the mTOR signaling pathway [23]. However, for mTOR activation, the functional LAT1 is coupled to ASCT2, another AA transporter involved in glutamine uptake [16].

The ASCT2 (SLC1A5) is expressed in most human tissues including the large intestine and CRC tumor cells, and is essentially responsible for the influx of glutamine inside the cells, inducing asparagine, serine and threonine efflux [24,25,26]. According to Liu and colleagues, ASCT2 expression levels can modulate the migration capacity of CRC cells, being the overexpression of this AA transportersassociated with a poorer patients’ prognosis [1,27]. In fact, ASCT2 is upregulated in several cancers, including triple-negative breast cancer, CRC, lung cancer, melanoma, neuroblastoma, glioblastoma and prostate cancer [12]. Some studies in glioblastomas and neuroblastoma support the involvement of the activation of c-Myc, n-Myc oncogenes in the inducing of ASCT2 expression [28,29].

**Figure 1:**
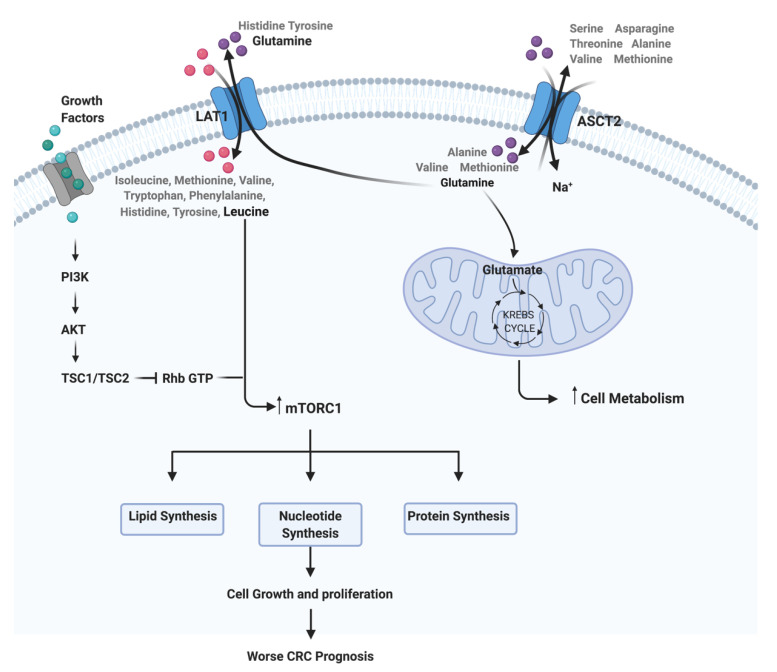
Representation of the interplay between ASCT2, LAT1 and mTOR pathway in colorectal cancer (CRC). This image was created using BioRender.

Metabolic reprogramming is a well-known hallmark of cancer that has been gaining increasing attention in the last few years due to its importance in cancer cells viability and growth [30]. Cancer associated metabolic reprogramming influences intracellular and extracellular availability of metabolites that will result in alterations in gene expression, cellular differentiation and also in the tumor microenvironment [31]. Glutamine is considered to be a crucial nutrient for cancer proliferation due to its ability to donate its nitrogen and carbon to several growth-promoting pathways [32]. In 2012, Mootha and colleagues reported that tumor cells have a high necessity of glutamine uptake compared to other AA and, consequently, a glutamine starvation can interfere with tumor metabolism inhibiting tumor proliferation and progression [32]. More recently, Varshavi and colleagues, described a molecular association between CRC that present oncogenic *KRAS* mutation and glutamine metabolism, since these cells exhibit special metabolic phenotypes, including differences in glycolysis, glutamine utilization and AA metabolism [33]. Furthermore, glutamine is described as a signaling factor in the uptake of AA for the activation of mTORC1 [34]. Thus, the upregulation of AA transporters have an important role in the support of the high-level protein synthesis for continuous cancer growth and proliferation [10,35]. The mTOR pathway is well described as deregulated in CRC, and the availability of AA functions as a regulator of this pathway, since a high AA microenvironmental bioavailability induces mTOR activity and consequent biological processes, such as protein translation [36]. Some studies report a relationship between LAT1 and ASCT2, with a two-step mechanism of these AAT being able to regulate mTOR pathway [37,38,39]. Firstly, ASCT2 regulates the intracellular concentration of glutamine, and in turn LAT1 uses this intracellular glutamine as an efflux substrate, in order to regulate the uptake of extracellular leucine, which will lead to an activation of mTOR signaling and consequent induction of cell growth and proliferation [40,41] (Figure 1). Furthermore, according to Rajasinghe and coworkers, the inhibition of glutamine uptake in proliferating cells, through the inhibition of glutamine transporters LAT1 and ASCT2, results in the inhibition of cell proliferation and induces apoptosis, through the downregulation of the mTOR pathway [38]. Thus, the inhibition of LAT1 and ASCT2 expression levels could represent a promising therapeutic approach for CRC since it would reduce the AA intake, consequently causing mTOR pathway inhibition and compromising cancer cell proliferation.

The use of pharmacologic approaches against LAT1 and ASCT2 in cancers with overexpression of these two AA transporters seems be a promising strategy. In fact, over the last few years there was investment in the development of drugs against LAT1 and ASCT2 [26,38,42,43]. The design of drugs against these two AA transporters usually follows an approach based on substrate analogues, which act as competitive inhibitors [26]. In the case of ASCT2 there are also been developed monoclonal antibodies against its cell surface domains [44]. The pharmacological inhibitors against LAT1 and ASCT2 reported in CRC are listed on Table 1.

More recently, in a phase I study, Okano and coworkers observed that the JPH203 treatment was well tolerated by patients with CRC and biliary tract cancer (BTC). In fact, disease control was observed in two of the six CRC patients and in three of the five BTC patients [13]. Furthermore, a study from Toda and colleagues using two *KRAS*-mutated cells lines demonstrated a significant association between ASCT2 expression and *KRAS* mutation and, when the authors used siRNAs to silence KRAS, they observed a significant reduction of ASCT2 [48]. In addition to that, the authors also used specific inhibitors of Raf/MEK/ERK, and PI3K/Akt/mTOR pathways, and observed that both inhibitors presented the ability to reduce ASCT2 expression [48]. Moreover, studies using xenograft models demonstrated that the inhibition of ASCT2 expression is able to reduce the uptake of glutamine and inhibit tumor cell proliferation [49]. However, it is imperative to keep in mind that the block of AA transporters could be associated with the upregulation of compensatory and redundant pathways, being crucial an accurate overview of all network involved in the process [50]. In addition to that, there are some limitations in the use of pharmacological inhibitors due to the low affinity for the transporter and low selective capacity observed to cancer cells. Thus, these data highlight the need for a deeper understanding of other therapeutic approaches for the selective inhibition of LAT1 and ASCT2 in CRC.

### 1.2. Applicability of microRNAs as Therapeutic Agents

Over the years, advances in genomic technologies have led to an identification of a variety of epigenetic alterations believed to be strongly involved in cancer initiation and progression [51]. In fact, several studies revealed that the altered metabolic pathways in cancer are tightly regulated by microRNAs (miRNAs) [52,53,54,55,56,57,58]. MiRNAs are a family of short non-coding RNAs with a length of approximately 19–25 nucleotides that post-transcriptionally regulate gene expression, with an important role in several biological pathways, including cell proliferation and differentiation [59,60]. MiRNAs can regulate the expression of more than 50% of protein-coding genes by binding to their target mRNA transcript and causing its degradation or translation repression [61]. Furthermore, the downstream targets of several miRNAs are directly or indirectly connected to metabolic alterations [52].

Regarding their applicability in the clinical setting, a growing number of evidence suggests a significant utility of miRNAs as biomarkers for pathogenic conditions, modulators of drug resistance and as therapeutic agents for medical intervention in almost all human health-related conditions [62,63,64,65]. The pleiotropic nature of miRNAs makes them particularly attractive, both as drugs or drug targets, for diseases with a multifactorial origin and no current effective treatments [66,67]. In addition to that, circulating miRNAs present several advantages compared to other circulating nucleic acids, such as: protection from RNAse degradation, high stability in circulation through the body, and resistance to adverse conditions such as temperature or pH alterations [68,69]. Regarding miRNA therapeutics applicability, there are reports demonstrating clinical utility of miRNA mimics and miRNA repressors and miRNAs loaded in something. In fact, there are two major types of miRNA-based therapies: miRNA suppression therapy, when the goal is the target mRNA upregulation and miRNA replacement therapy, when the goal is the target mRNA downregulation.

**Figure 2:**
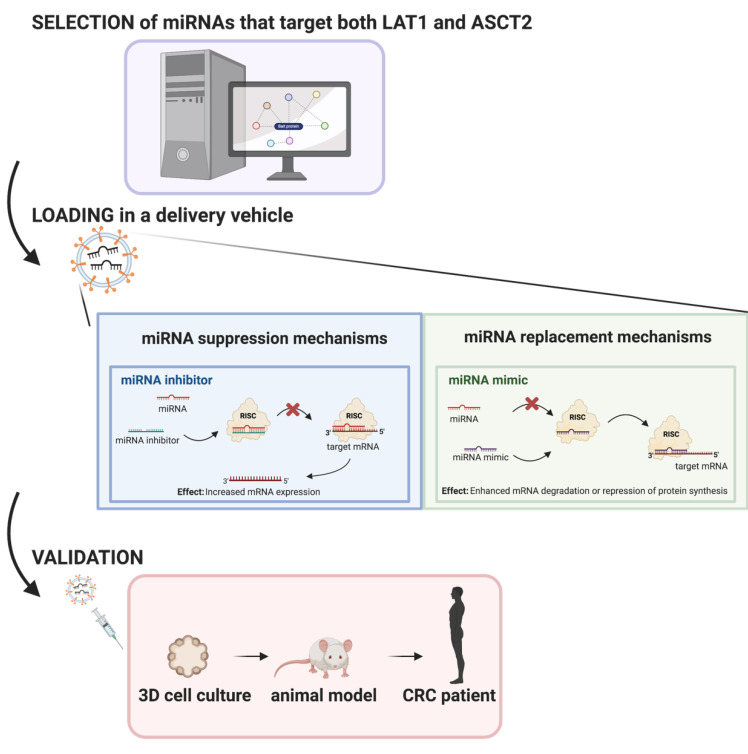
Schematic overview of the applicability of this review in the future development of miRNA-based therapies against LAT1 and ASCT2 in CRC. This image was created using BioRender.

Overall, the current evidence suggests a viable future for miRNA drugs in diseases with no current effective treatments, such as CRC. Hence, the scope of this review is to gather and systematize the information available regarding the impact of *LAT1* and *ASCT2* related miRNAs in CRC development and establish a profile with potential application to be used as a therapeutic agent through in silico analysis combined with a literature review (Figure 2).

## 2. Materials and Methods

### 2.1. MiRNA Selection and Literature Review

**Figure 3:**
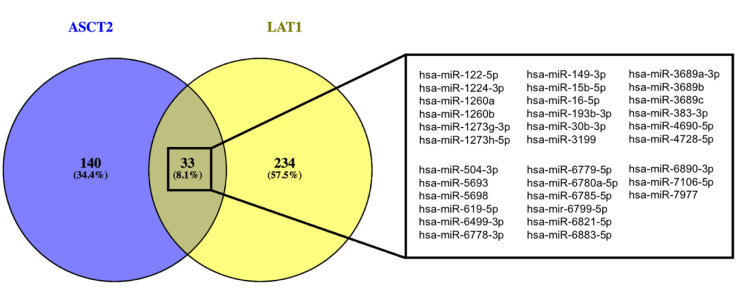
Venn diagram of the validated miRNAs that target ASCT2 and LAT1 obtained using Venny 2.1. (http://bioinfogp.cnb.csic.es/tools/venny, accessed on 21 December 2020) and the detailed list of the 33 miRNAs.

In order to select miRNAs that target both *LAT1* and *ASCT2* we used miRTarBase (version 8.0), the largest known online database of validated miRNA:mRNA interactions [70]. According to miRTarBase there are 267 miRNAs that target *LAT1* and 173 that target *ASCT2* mRNAs. Since one miRNA has multiple targets and the same mRNA can be regulated by several miRNAs, we went to see if there were miRNAs that targeted both *LAT1* and *ASCT2*. From the 440 miRNAs retrieved by miRTarBase, we observed that 33 targeted both *LAT1* and *ASCT2* (Figure 3).

After retrieving the miRNAs that target both *LAT1* and *ASCT2* from miRTarBase, a literature search in PubMed and Google Academic was conducted using the search terms “colorectal cancer” plus one of the following 33 microRNAs: “miR-122-5p”, “miR-1224-3p”, “miR-1260a”, “hsa-miR-1260b”, “hsa-1273g-3p”, hsa-miR-1273h-5p”, “hsa-miR-149-3p”, “hsa-miR-15b-5p”, “miR-16-5p”, “miR-193b-3p”, “miR-30b-3p”, “miR-3199”, “miR-3689a-3p”, “miR-3689b-3p”, “miR-3689c”, “miR-383-3p”, “miR-4690-5p”, “miR-4728-5p”, “miR-504-3p”, “miR-5693”, “miR-5698”, “miR-619-5p”, “miR-6499”, “miR-6778-3p”, “miR-6799-5p”, “miR-6780a-5p”, “miR-6785”, “miR-6799”, “miR-6821”, “miR-6883-5p”, “miR-6890-3p”, “miR-7106-5p” or “miR-7977”. The articles were selected by relevance of their findings, namely, a significant association between these miRNAs and colorectal cancer. Literature analysis includes scientific papers published in the last 6 years (between 2014 and 2020). The obtained scientific papers were manually curated in order to determine associations between the miRNAs and CRC, giving a total of 28 selected papers. The exclusion criteria for the collected papers were as follows: (1) no significant association between the miRNAs and CRC; (2) association of the miRNAs with a benign tumor and (3) individual papers that were already included in meta-analysis. For each study, information was extracted concerning the following characteristics: the name of the miRNA, type of sample where the miRNA was studied, miRNA expression levels (upregulated and downregulated) and their effect on CRC (e.g., prognosis, therapy response or pathways regulation).

### 2.2. In Silico Analysis

The Search Tool for the Retrieval of Interacting Genes (STRING) database is an online tool that is used to develop protein–protein interaction (PPI) networks [71]. We used the STRINGapp of the Cytoscape software (v3.7.X) to construct and visualize the protein interaction network of the selected target genes. Those with a combined score of >0.4 were selected as significant. The functional enrichment analysis of Gene Ontology (GO), Kyoto Encyclopedia of Genes and Genomes (KEGG) and Reactome pathways was made with the STRING enrichment analysis tool, with a false discovery rate (FDR) of *p* < 0.01. The enrichment results were filtered, and redundant terms were removed according to the Jaccard index.

## 3. Results

### 3.1. miRNAs that Target Both LAT1 and ASCT2 and their Impact on CRC

From the 33 candidate miRNAs, only 16 have already been described in CRC (Table 2). However, in terms of the miRNA: mRNA target interaction with *LAT1* and *ASCT2*, none of the miRNAs have been yet validated for CRC.

Through the analysis of Table 1 we can observe that some of the miRNAs present opposite results regarding their expression levels, which may be related with the type of biological sample from which their expression levels are analyzed. Regarding their effects on CRC, the deregulation of miR-122-5p, miR-1273g-3p, miR-16-5p, miR-3199, miR-383-3p, miR-619-5p and miR-6883-5p was associated with the upregulation of important players of oncogenic pathways, such as TRIM29, CDC25A, PI3K/Akt, mTOR, VEGFA, MALAT1, SMAD4, STMN1, APRIL and CDK4, with an impact on cell proliferation, invasion and migration. In addition to that, miR-1260b, miR-149-3p and miR-15b-5p were reported as associated with resistance to 5′-FU treatment through the upregulation of PDCD4, PDK2 and XIAP, respectively. Moreover, only three miRNAs were associated with clinical endpoints. Higher plasmatic levels of hsa-miR-122-5p were associated with worse prognosis in metastatic patients and shorter RFS and OS in non-metastatic patients, while lower levels of CRC tissue hsa-miR-193b-3p and hsa-miR-619-5p were associated with shorter OS. Moreover, lower levels of CRC tissue hsa-miR-619-5p were also associated with shorter DFS, lymphovascular invasion and perineural invasion.

### 3.2. Functional Annotation and Pathway Enrichment Analysis

Since the downregulation of a miRNA usually leads to the upregulation of its mRNA targets, we focused on the 11 miRNAs that have been reported as downregulated in CRC cells and tissues and therefore could be implicated in the upregulation of *LAT1* and *ASCT2* (miR-122-5p, miR-1260b, miR-149-3p, miR-15b-5p, miR-16-5p, miR-193b-3p, miR-3199, miR-383-3p, miR-619-5p, miR-6821-5p and miR-6883-5p) and did an in silico analysis to obtain a deeper knowledge of their impact on CRC. We used miRTarBase v8.0 to retrieve the mRNA targets of the selected miRNAs that were validated with strong evidence methods in order to do the functional annotation and enrichment analysis. From the 11 miRNAs studied, only miR-15b-5p, miR-16-5p, miR-122-5p, miR-149-3p, miR-1260b, miR-193b-3p and miR-383-3p presented mRNA targets validated with strong evidence methods (Western blot, qRT-PCR or luciferase assay), which are listed on Table 3.

**Figure 4:**
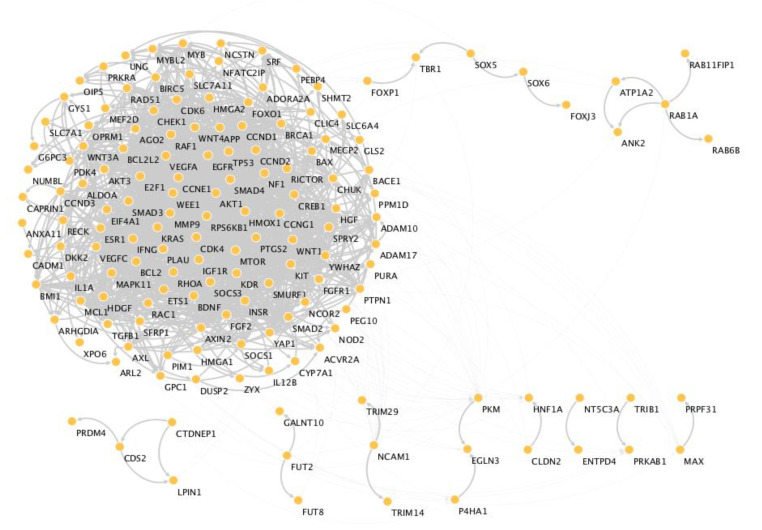
Protein–protein interaction (PPI) network. The proteins were clustered using the clusterMaker app from Cytoscape with an inflation value of 2.5 and a cutoff edge of 0.5. All the singletons were removed.

In order to explore the biological impact of these miRNA profiles in CRC, we analyzed their 186 validated targets with the STRINGapp Protein Query from Cytoscape software. A total of 168 of the 186 coding genes were filtered into a protein–protein interaction (PPI) network with 168 nodes and 1284 edges that presented a significant enrichment (*p* = 1 × 10^−16^). We also applied a Markov clustering (MCL), which resulted in the clustering of the proteins into 11 clusters according to their STRING interaction score (Figure 4).

**Figure 5:**
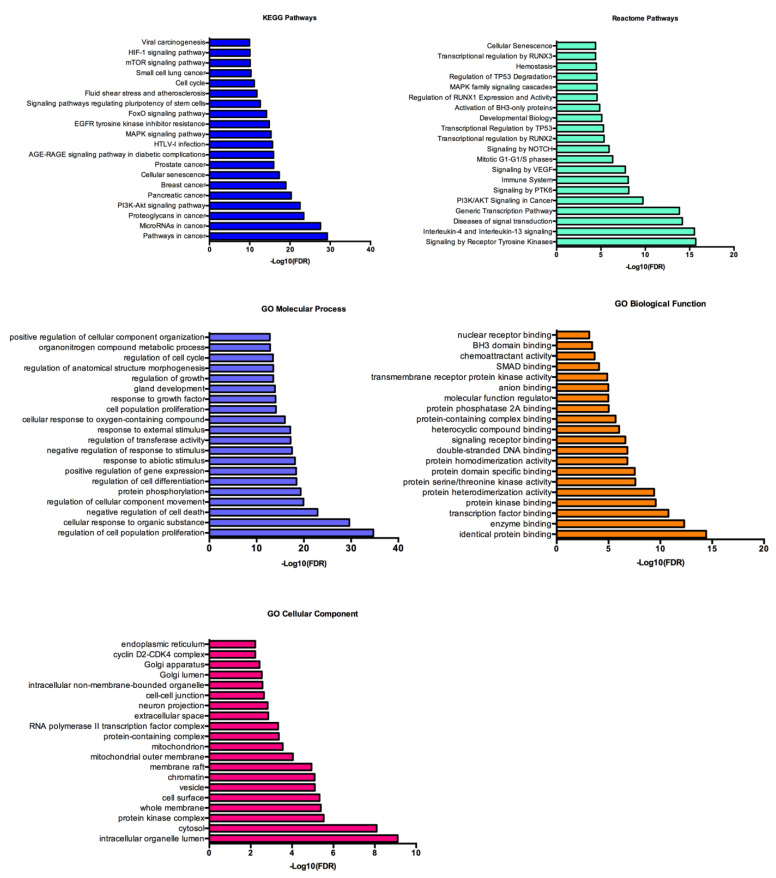
Kyoto Encyclopedia of Genes and Genomes (KEGG), Reactome and Gene Ontology (GO) analysis of the 186 selected target genes. The functional enrichment analysis was made using the STRINGapp from Cytoscape.

The functional enrichment analysis was made using an FDR threshold of *p* < 0.01, and the redundant terms were eliminated using a redundancy cutoff of 0.5, which resulted in a total of 892 enriched terms among the KEGG, Reactome and GO categories (Supplementary Tables S1–S5). The top 20 enriched terms for each category are represented on Figure 5. Among the functionally enriched terms in the KEGG and Reactome pathways we could find PI3K/Akt, MAPK, HIF-1, mTOR, VEGF and EGFR inhibitor resistant pathways, all of which are well established as involved in CRC development. Regarding the GO terms, if we focus on the molecular processes, we can observe that the two most enriched terms are the regulation of cell proliferation and the cellular response to organic substances, which may be related with the increase intake of nutrients as a consequence of the downregulation of this miRNA profile and consequent upregulation of AA transporters, such as LAT1 and ASCT2.

## 4. Discussion

CRC remains one of the most diagnosed cancers in the world, with a high metastatic potential and not enough therapeutic options. The previous underestimated metabolic alterations are now gaining more attention from the scientific community, and it is now known that metabolic cross-communication between tumor cells, immune cells, stromal cells and the gut microbiota are able to induce CRC proliferation, invasion and metastasis [98]. Among the metabolic alterations with potential to be targeted in order to develop new therapeutic approaches, the upregulation of AA transporters *LAT1* and *ASCT2* seems promising due to their impact in the regulation of the mTOR pathway. In addition to that, the liver is recognized as the most common metastatic site of CRC and cumulative evidences show that LAT1 and ASCT2 are overexpressed in hepatocellular carcinoma (HCC) and that these cells present a 10–20 fold increase in glutamine uptake, compared to normal hepatocytes [27,38,99,100,101]. Therefore, the definition of a new therapeutic approach involving the inhibition of these two AA transporters could be a promising strategy to control CRC proliferation and aggressiveness.

Recently, it has been suggested that modulation of miRNAs in cancer cells could be a potential tool for the improvement of cancer patients’ therapies. In fact, by suppressing oncogenic miRNAs or substituting deficient tumor suppressive miRNAs, we are able to control cancer cell growth and progression. The world’s first miRNA therapeutic, a short locked nucleic acid (LNA) antagonist for miR-122 named Miravirsen (produced by Roche/Santaris) was developed for the treatment of hepatitis C virus (HCV) infection [102]. Along with Miravirsen, all of the miRNA-based drugs are currently in clinical trials and none have yet reached the pharmaceutical breakthrough. However, acquisition of miRNA-based companies by famous pharmaceutical companies is sending a positive feedback on their potential [103]. Currently, there are several strategies used for miRNA-based therapies, which could include miRNA inhibition therapies that target oncomiRNAs, replacement therapies for tumor-suppressor miRNAs or miRNA-based delivery systems [104]. One example, of their applicability was the study performed by Callegari and coworkers that showed that the in vivo delivery of an anti-miR-221 caused a significant decrease in the size and number of tumor nodules, being established the promotor role of miR-221 in liver carcinogenesis [105]. On the other hand, Oshima and coworkers reported an effective delivery of miR-655-3p to CRC liver metastasis using nanoscale coordination polymers. The polymers used prolonged the miRNA distribution and miRNA-655-3p suppressed tumor growth when codelivered with oxaplatin, suggesting a synergistic effect of both therapeutic approaches [106].

Regarding the delivery mechanisms, miRNAs are delivered through the use of vectors that can be divided into two categories: viral vectors and nonviral vectors. The viral vectors used for miRNA delivery are mainly adenovirus vectors, adeno-associated virus vectors, retroviral vectors and lentivitus vectors. On the other hand, nonviral vectors include inorganic material-based delivery systems, lipid-based nanocarriers, polymeric vectors/dendrimer-based vectors, cell-derived membrane vesicles and 3D-Scaffold-based delivery systems [107]. The use of these delivery mechanisms improves targeting ability while protecting the miRNAs or miRNAs inhibitors from degradation. In fact, it was already demonstrated that, for cancer treatment, intratumoral injections of miRNA drugs directly into the tumor site are able to enhance target efficacy, specificity and minimize the side effects and there are also several ongoing clinical trials [62,108,109].

In the present review we combined a bioinformatic approach with a literature review to define a miRNA profile (miR-15b-5p, miR-16-5p, miR-122-5p, miR-1260b, miR-149-3p, miR-193b-3p and miR-383-3p) that has the potential to target both LAT1 and ASCT2 in CRC. The in silico approaches are very useful since they allow the simultaneous analysis of the interactions of hundreds of genes and, therefore, the creation of an integrative network that allows a deeper understanding of the biological processes regulated by them. Our in silico analysis result in a list of miRNAs that target both LAT1 and ASCT2 and the literature review allowed us to focus on the miRNAs that have already been studied in CRC and have been reported as downregulated. In addition to that, functional enrichment analysis showed that among the enriched terms derived from the miRNA profile targets, we can find PI3K/Akt, MAPK, HIF-1, mTOR, VEGF and EGFR inhibitor resistant pathways, all of which are well established as involved in CRC development.

The MAPK and PI3K/Akt signaling pathways are involved in cell proliferation and survival, and their deregulation confers proliferative advantages on cancer cells. In fact, KRAS, BRAF and PI3K mutations are frequent in CRC. Moreover, the increase of the PI3K/Akt pathway activation in CRC is also associated with the loss of the tumor suppressor PTEN, which is significantly associated with a worse prognosis [1]. In addition to that, according to Slatery and colleagues, approximately 41% of the genes of MAPK signaling are dysregulated in CRC [110]. These two signaling cascades can activate directly and indirectly the Ser/Thr protein kinase mTOR, respectively, being the mTOR involved in the regulation of cell proliferation and survival [111]. Similarly to other solid tumors, CRC is also characterized by a hypoxic microenvironment [112]. In fact, the cancer cells have the ability of adaptation to hypoxia through the regulation of the PI3K/AKT/mTOR pathway and by the transcription factors HIF-1α and HIF-2α, whose protein expression and transcriptional activity are also regulated by mTOR [113]. Furthermore, tumor hypoxia can also enhance cancer cells survival and proliferation through the upregulation of VEGF and its receptor VEGFR. VEGF promotes CRC growth through the stimulation of angiogenesis and its downstream signaling pathways are well characterized in cancer, with VEGF/VEGFR activation leading to the activation of MAPK/ERK, PI3K/Akt, PLC/PKC and other signaling pathways [114,115] .

Taking this information into consideration, we can conclude that the miRNA profile proposed in the present study plays an important role on CRC development and aggressiveness. However, despite promising, the results are still preliminary and require validation in CRC study models in order to assess the miRNA profile interaction with LAT1 and ASCT2 mRNAs, especially in terms of its inhibitory power. During the past few years, there has been a significant development of in vitro and in vivo preclinical research models, such as 3D cell culture of spheroids and organoids derived from several human tissues, which is helping in the translation of miRNAs into clinical practice [66,116,117]. In a recent study, Kawai and colleagues determined the culture conditions necessary to establish 3D cell culture models that mimic colon cancer heterogeneity [118]. In another study, Zoetemelk and colleagues established a robust, low-cost and reproducible short-term 3D colorectal cancer spheroids model to be used as a platform for screening the effect of combination therapies in CRC [119]. These enhanced research models are very useful for the study of miRNAs dynamics and for the development of the delivery systems for miRNA-based therapeutics [120,121]. Therefore, the next step should be focused on the delivery of the miRNA profile to CRC 3D culture models in order to see if it is sufficient to reverse the increased AA uptake caused by the increase of LAT1 and ASCT2 and inhibit cell proliferation.

## Figures and Tables

**Table 1:** Pharmacological inhibitors of LAT1 and ASCT2 reported in CRC.

AA Transporter	Inhibitor	Inhibitor Type	Reference
LAT1	JHP203	Tyrosine analog	[45,46]
ASCT2	MAb KM4008	Monoclinal antibodies against cell surface domains	[44]
	MAb KM4012		
	MAb KM4018		
	V-9302	Competitive antagonist	[47]

**Table 2:** Selected miRNAs’ impact on CRC.

miRNA	Expression	Sample Type	Effect	Reference
Hsa-miR-122-5p	Down	CRC Tissue and cells	Increase in cell proliferation, migration and invasion through the upregulation of CDC25A	Yin 2020 [72]
	Down	CRC Tissues	Upregulation of the PI3K/Akt pathway through upregulation of TRIM29	Asadi 2019 [73]
	Up	CRC liver metastatic tissues	Not described	Liu 2019 [74]
	Up	Serum and HT-29 and SW480 cell lines	Lymph node metastasis biomarker and cell migration inducer	Qu 2018 [75]
	Up	CRC Plasma	Worse prognosis in metastatic patients and shorter RFS and OS in non-metastatic patients	Maiertheler 2017 [76]
Hsa-miR-1224-3p	Up	CRC Tissues	Upregulated in E cadherin positive tissues	Lin 2017 [77]
Hsa-miR-1260a	Down	CRC Serum	Not described	Wang 2017 [78]
Hsa-miR-1260b	Up	HCT116 cells	Chemoresistance to 5-FU through upregulation of PDCD4	Zhao 2018 [79]
	Down	SW480 cells	Downregulated by STAT3-siRNA	Zhang 2014 [80]
	Up	Carcinoma vs adenoma (tissue)	Not described	Slattery 2016 [81]
	Down	CRC Serum	Not described	Zhang 2017 [82]
	Up	DKO-1 cells	Enriched in *KRAS* mutant cells	Cha 2015 [83]
Hsa-miR-1273g-3p	Up	LoVo cells	Proliferation, migration and invasion through activation of ERBB4/PIK3R3/mTOR/S6K2 pathway	Li 2018 [84]
Hsa-miR-1273h-5p	Up	CRC tissues	Not described	Du 2018 [85]
Hsa-miR-149-3p	Down	HCT-8 and HCT-116 cells	Chemoresistance to 5-FU through upregulation of PDK2	Liang 2020 [86]
Hsa-miR-15b-5p	Down	CRC tissues and cell lines	Chemoresistance to 5-FU through upregulation of XIAP	Zhao 2017 [87]
	Up	HT-29 cell line	Cell growth and inhibition of the proapoptotic pathway	Gasparello 2020 [88]
	Down	KRAS mutated CRC tissues vs wild type CRC tissues	Not described	Milanesi 2020 (82)
Hsa-miR-16-5p	Down	CRC tissues and cell lines	Upregulation of VEGFA	Wu 2020 [33]
Hsa-miR-193b-3p	Down	CRC tissues vs adjacent normal tissues	Shorter OS of CRC patients and upregulation of STMN1	Guo 2016 [89]
	Up	CRC tissues	Downregulation of RAD51	Kara 2015 [90]
Hsa-miR-3199	Down	SW620 cell line	Upregulation of SMAD4	Yan 2018 [91]
Hsa-miR-383-3p	Down	CRC tissues and HT-29 and LoVo cell lines	Upregulation of APRIL	Cui 2018 [92]
Hsa-miR-4690-5p	Down	CRC Stool	Not described	Ghanbari 2015 [93]
	Up	CRC tissues	Upregulated in CIMP high/MSI CRC tissues	Mullany 2016 [94]
Hsa-miR-619-5p	Down	CRC tissues vs adjacent normal tissues	Upregulation of MALAT1, lymphovascular invasion perineural invasion, shorter DFS and shorter OS	Qiu 2016 [95]
Hsa-miR-6821-5p	Down	SW480 CSCs vs SW480 wild-type	Not described	Zhou 2019 [96]
	Up	CRC tissues	Not described	Du 2018 [85]
Hsa-miR-6883-5p	Down	TCGA dataset and Cell lines	Upregulation of CDK4 and CDK6 and cell growth stimulus	Lulla 2017 [97]

**Table 3:** Validated targets of miR-15b-5p, miR-16-5p, miR-122-5p, miR-1260b, miR-149-3p, miR-193b-3p and miR-383-3p.

miRNA	Target mRNA
miR-16-5p	*ZYX, YAP1, WNT4, WNT3A, WEE1, VEGFA, UNG, UCA1, TPPP3, TP53, SOX6, SOX5, SOCS3, SLC6A4, RPS6KB1, RICTOR, RECK, RAF1, PURA, PTGS2, PRDM4, PPM1D, PIM1, OPRM1, NCSTN, NCOR2, MYB, MTOR, METTL13, MAP7, KRAS, KDR, IL12B, IGF1R, IFNG, HMGA2, HMGA1, HGF, HDGF, GLS2, FGFR1, FGF2, CLDN2, CHUK, CHEK1, CDS2, CDK6, CCNE1, CCND3, CCND2, CCND1, CAPRIN1, CADM1, BRCA1, BMI1, BIRC5, BDNF, BCL2, BACE1, AXIN2, ARL2, ARHGDIA, APP, AKT3, ADORA2A, ACVR2A*
miR-15b-5p	*WEE1, VEGFA, TRIM29, TRIM14, TGFB1, TBR1, SOCS3, SMURF1, SMAD2, RECK, RAB1A, PURA, PPM1D, PEBP4, OIP5, MTSS1, MMP9, KDR, INSR, IFNG, HNF1A, FUT2, FOXO1, EIF4A1, CHEK1, CCNE1, CCND3, CCND1, BCL2, BAX, AXIN2, AKT3, AGO2*
miR-122-5p	*ZNF395, XPO6, WNT1, VEGFC, UBAP2, TRIB1, TPD52L2, TBX19, SRF, SPRY2, SOCS1, SLC7A11, SLC7A1, RHOA, RAC1, RAB6B, RAB11FIP1, PTPN1, PRKRA, PRKAB1, PKM, PEG10, PDK4, P4HA1, NUMBL, NT5C3A, NOD2, NFATC2IP, NCAM1, MEF2D, MECP2, MAPK11, LPIN1, IL1A, IGF1R, HMOX1, GYS1, GALNT10, G6PC3, FUT8, FUNDC2, FOXP1, FOXJ3, FAM117B, ENTPD4, EGLN3, EGFR, DUSP2, DSTYK, CYP7A1, CTDNEP1, CREB1, CLIC4, CDK4, CCNG1, BCL2L2, BAX, AXL, ATP1A2, AP3M2, ANXA11, ANK2, ALDOA, AKT3, ADAM17, ADAM10, AACS*
miR-1260b	SMAD4, SFRP1, DKK2
miR-193b-3p	*YWHAZ, SMAD3, SHMT2, RAD51, PRAP1, PLAU, NF1, MYB, MCL1, MAX, KRAS, KIT, ETS1, ESR1, CCND1, AKR1C2*
miR-383-3p	*PRPF31*
miR-149-3p	*WNT1, MYBL2, GPC1, FGFR1, E2F1, AKT1*

## Supplementary Materials

The following are available online at https://www.mdpi.com/2227-9059/9/2/195/s1, Table S1: GO Cellular Component enrichment results for terms with FDR *p* < 0.01, Table S2: GO Molecular Function enrichment results for terms with FDR *p* < 0.01, Table S3: GO Biological Process enrichment results for terms with FDR *p* < 0.01, Table S4: KEGG pathway enrichment results for terms with FDR *p* < 0.01, Table S5: Reactome pathways enrichment results for terms with FDR *p* < 0.01.
