# Supplementary material for: LAT1 and ASCT2 Related microRNAs as Potential New Therapeutic Agents against Colorectal Cancer Progression
Source: Biomedicines. 2021 Feb 16;9(2):195. doi: 10.3390/biomedicines9020195 (PMC7920065; doi:10.3390/biomedicines9020195)
Supplement: Supplementary file 1 [file biomedicines-09-00195-s001.pdf]

Supplementary material

Table S1. GO Cellular Component enrichment results for terms with FDR *P*<0.01

Number of Genes	Term name	Description	FDR
86	GO.0070013	intracellular organelle lumen	7.79E-10
81	GO.0005829	cytosol	8.09E-9
9	GO.1902911	protein kinase complex	2.92E-6
35	GO.0098805	whole membrane	4.12E-6
22	GO.0009986	cell surface	4.63E-6
44	GO.0031982	vesicle	7.96E-6
18	GO.0000785	chromatin	8.09E-6
14	GO.0045121	membrane raft	1.15E-5
10	GO.0005741	mitochondrial outer membrane	9.03E-5
30	GO.0005739	mitochondrion	2.8E-4
65	GO.0032991	protein-containing complex	4.4E-4
8	GO.0090575	RNA polymerase II transcription factor complex	4.7E-4
23	GO.0005615	extracellular space	0.0014
23	GO.0043005	neuron projection	0.0015
12	GO.0005911	cell-cell junction	0.0023
54	GO.0043232	intracellular non-membrane-bounded organelle	0.0027
6	GO.0005796	Golgi lumen	0.0029
26	GO.0005794	Golgi apparatus	0.0037
2	GO.0097129	cyclin D2-CDK4 complex	0.006
29	GO.0005783	endoplasmic reticulum	0.006
5	GO.0070062	extracellular exosome	0.0063
21	GO.0031967	organelle envelope	0.007
9	GO.0005788	endoplasmic reticulum lumen	0.0093

Table S2. GO Molecular Function enrichment results for terms with FDR *P*<0.01

Number of Genes	Term name	Description	FDR
55	GO.0042802	identical protein binding	3.76E-15
58	GO.0019899	enzyme binding	5.04E-13
29	GO.0008134	transcription factor binding	1.65E-11
27	GO.0019901	protein kinase binding	2.75E-10
25	GO.0046982	protein heterodimerization activity	3.94E-10
21	GO.0004674	protein serine/threonine kinase activity	2.57E-8
26	GO.0019904	protein domain specific binding	2.92E-8
27	GO.0042803	protein homodimerization activity	1.51E-7
27	GO.0003690	double-stranded DNA binding	1.51E-7
37	GO.0005102	signaling receptor binding	2.36E-7
79	GO.1901363	heterocyclic compound binding	9.41E-7
27	GO.0044877	protein-containing complex binding	2.06E-6
6	GO.0051721	protein phosphatase 2A binding	9.46E-6
37	GO.0098772	molecular function regulator	1.02E-5
48	GO.0043168	anion binding	1.02E-5
8	GO.0019199	transmembrane receptor protein kinase activity	1.33E-5
7	GO.0046332	SMAD binding	8.12E-5
5	GO.0042056	chemoattractant activity	2.2E-4
3	GO.0051434	BH3 domain binding	3.8E-4
7	GO.0016922	nuclear receptor binding	7.0E-4
3	GO.0070878	primary miRNA binding	9.1E-4
14	GO.0003682	chromatin binding	0.0013
6	GO.0042562	hormone binding	0.0015
14	GO.0003712	transcription coregulator activity	0.0021
8	GO.0019887	protein kinase regulator activity	0.0027
4	GO.0001221	transcription cofactor binding	0.0029
5	GO.0033613	activating transcription factor binding	0.0033
10	GO.0031625	ubiquitin protein ligase binding	0.0034
6	GO.0042826	histone deacetylase binding	0.0037
6	GO.0005178	integrin binding	0.0057
2	GO.0051425	PTB domain binding	0.0073
3	GO.0005158	insulin receptor binding	0.0091
2	GO.0043559	insulin binding	0.0097

Table S3. GO Biological Process enrichment results for terms with FDR *P*<0.01

Number of Genes	Term name	Description	FDR
78	GO.0042127	regulation of cell population proliferation	1.89E-35
83	GO.0071310	cellular response to organic substance	2.33E-30
51	GO.0060548	negative regulation of cell death	1.23E-23
46	GO.0051270	regulation of cellular component movement	1.21E-20
46	GO.0006468	protein phosphorylation	4.66E-20
59	GO.0045595	regulation of cell differentiation	3.6E-19
61	GO.0010628	positive regulation of gene expression	4.23E-19
47	GO.0009628	response to abiotic stimulus	7.57E-19
54	GO.0048585	negative regulation of response to stimulus	3.16E-18
44	GO.0051338	regulation of transferase activity	6.31E-18
64	GO.0009605	response to external stimulus	7.42E-18
41	GO.1901701	cellular response to oxygen-containing compound	1.04E-16
34	GO.0008283	cell population proliferation	7.89E-15
30	GO.0070848	response to growth factor	9.68E-15
27	GO.0048732	gland development	1.25E-14
33	GO.0040008	regulation of growth	2.89E-14
39	GO.0022603	regulation of anatomical structure morphogenesis	2.96E-14
42	GO.0051726	regulation of cell cycle	3.38E-14
94	GO.1901564	organonitrogen compound metabolic process	1.36E-13
41	GO.0051130	positive regulation of cellular component organization	1.54E-13
49	GO.0009888	tissue development	2.03E-13
32	GO.0008285	negative regulation of cell population proliferation	2.1E-13
15	GO.0043535	regulation of blood vessel endothelial cell migration	3.54E-13
29	GO.0009611	response to wounding	3.62E-13
23	GO.0050678	regulation of epithelial cell proliferation	3.83E-13
25	GO.2001233	regulation of apoptotic signaling pathway	4.63E-13
32	GO.0007167	enzyme linked receptor protein signaling pathway	5.8E-13
43	GO.0080134	regulation of response to stress	5.85E-13
36	GO.1902533	positive regulation of intracellular signal transduction	3.26E-12
14	GO.0048145	regulation of fibroblast proliferation	3.26E-12
30	GO.0010942	positive regulation of cell death	5.24E-12
21	GO.1901214	regulation of neuron death	6.44E-12
89	GO.0016043	cellular component organization	9.06E-12
34	GO.0009790	embryo development	1.03E-11
45	GO.0033554	cellular response to stress	1.06E-11
17	GO.2000377	regulation of reactive oxygen species metabolic process	1.15E-11
26	GO.0071407	cellular response to organic cyclic compound	1.5E-11
19	GO.0050730	regulation of peptidyl-tyrosine phosphorylation	1.91E-11
28	GO.0035239	tube morphogenesis	2.85E-11
15	GO.0016202	regulation of striated muscle tissue development	3.29E-11
25	GO.0009991	response to extracellular stimulus	4.12E-11
21	GO.0001101	response to acid chemical	4.44E-11
19	GO.0090287	regulation of cellular response to growth factor stimulus	5.42E-11
41	GO.0002682	regulation of immune system process	7.85E-11
27	GO.0030155	regulation of cell adhesion	2.0E-10
45	GO.0051049	regulation of transport	3.28E-10
14	GO.0048660	regulation of smooth muscle cell proliferation	3.5E-10
30	GO.0072359	circulatory system development	4.67E-10
20	GO.0043405	regulation of MAP kinase activity	6.45E-10
53	GO.0002376	immune system process	7.39E-10
15	GO.0048639	positive regulation of developmental growth	8.65E-10
14	GO.0033135	regulation of peptidyl-serine phosphorylation	8.65E-10
34	GO.0051248	negative regulation of protein metabolic process	1.19E-9
31	GO.0042493	response to drug	1.25E-9
27	GO.0045596	negative regulation of cell differentiation	1.34E-9
16	GO.2000027	regulation of animal organ morphogenesis	1.69E-9
27	GO.0060322	head development	1.73E-9
13	GO.0035270	endocrine system development	2.27E-9

25	GO.0002520	immune system development	2.54E-9
14	GO.0051897	positive regulation of protein kinase B signaling	4.46E-9
12	GO.0001952	regulation of cell-matrix adhesion	6.48E-9
35	GO.1903507	negative regulation of nucleic acid-templated transcription	6.68E-9
39	GO.0042592	homeostatic process	7.23E-9
13	GO.0048754	branching morphogenesis of an epithelial tube	7.31E-9
17	GO.0090068	positive regulation of cell cycle process	8.7E-9
16	GO.0007611	learning or memory	9.55E-9
13<br>29	GO.0071560<br>GO.0022402	cellular response to transforming growth factor beta stimulus<br>cell cycle process	1.15E-8<br>1.59E-8
32	GO.0007267	cell-cell signaling	1.59E-8
17	GO.1901653	cellular response to peptide	1.89E-8
14	GO.1904018	positive regulation of vasculature development	1.94E-8
17	GO.0009416	response to light stimulus	1.96E-8
26	GO.0050767	regulation of neurogenesis	2.14E-8
36	GO.0022414	reproductive process	2.18E-8
12	GO.0001889	liver development	2.93E-8
15	GO.0042110	T cell activation	3.58E-8
13	GO.0030307	positive regulation of cell growth	4.36E-8
15	GO.0045165	cell fate commitment	4.63E-8
21	GO.0032103	positive regulation of response to external stimulus	5.49E-8
27	GO.0048646	anatomical structure formation involved in morphogenesis	6.12E-8
10	GO.0031100	animal organ regeneration	6.54E-8
12	GO.0045834	positive regulation of lipid metabolic process	7.22E-8
37	GO.0048468	cell development	7.68E-8
15	GO.0031334	positive regulation of protein complex assembly	8.02E-8
28	GO.0006915	apoptotic process	1.05E-7
14	GO.0043281	regulation of cysteine-type endopeptidase activity involved in apoptotic process	1.09E-7
10	GO.0001657	ureteric bud development	1.32E-7
10	GO.0000079	regulation of cyclin-dependent protein serine/threonine kinase activity	1.32E-7
11	GO.0007498	mesoderm development	1.52E-7
20	GO.0010035	response to inorganic substance	2.09E-7
31	GO.0040011	locomotion	2.22E-7
11	GO.0045598	regulation of fat cell differentiation	2.23E-7
18	GO.1904951	positive regulation of establishment of protein localization	2.37E-7
10	GO.0097191	extrinsic apoptotic signaling pathway	2.5E-7
13	GO.0050920	regulation of chemotaxis	2.5E-7
10	GO.0090263	positive regulation of canonical Wnt signaling pathway	2.72E-7
26	GO.0018193	peptidyl-amino acid modification	3.0E-7
13	GO.0048872	homeostasis of number of cells	3.28E-7
22	GO.0001817	regulation of cytokine production	3.39E-7
32	GO.0051336	regulation of hydrolase activity	3.54E-7
14	GO.0097305	response to alcohol	3.6E-7
31	GO.0051707	response to other organism	3.69E-7
20	GO.1902532	negative regulation of intracellular signal transduction	4.02E-7
20	GO.0044057	regulation of system process	4.23E-7
11	GO.0008584	male gonad development	5.12E-7
8	GO.0035019	somatic stem cell population maintenance	5.87E-7
15	GO.0040013	negative regulation of locomotion	5.89E-7
10	GO.2001237	negative regulation of extrinsic apoptotic signaling pathway	6.06E-7
17	GO.0051052	regulation of DNA metabolic process	6.82E-7
12	GO.2001235	positive regulation of apoptotic signaling pathway	6.99E-7
14	GO.0051091	positive regulation of DNA-binding transcription factor activity	7.63E-7
13	GO.0044706	multi-multicellular organism process	8.01E-7
16	GO.0048589	developmental growth	8.35E-7
13	GO.0001503	ossification	9.67E-7
9	GO.0070542	response to fatty acid	9.72E-7
72	GO.0009058	biosynthetic process	9.73E-7

7	GO.0090049	regulation of cell migration involved in sprouting angiogenesis	9.93E-7
9	GO.0050673	epithelial cell proliferation	1.05E-6
14	GO.1902105	regulation of leukocyte differentiation	1.2E-6
9	GO.1901216	positive regulation of neuron death	1.25E-6
11	GO.0051147	regulation of muscle cell differentiation	1.36E-6
11	GO.0010821	regulation of mitochondrion organization	1.36E-6
18	GO.0001501	skeletal system development	1.49E-6
19	GO.0010817	regulation of hormone levels	1.55E-6
18	GO.0090066	regulation of anatomical structure size	1.81E-6
18	GO.1903827	regulation of cellular protein localization	1.85E-6
9	GO.1901655	cellular response to ketone	1.85E-6
11	GO.0010001	glial cell differentiation	1.89E-6
18	GO.0051301	cell division	3.1E-6
15	GO.0062012	regulation of small molecule metabolic process	3.15E-6
16	GO.0003013	circulatory system process	3.2E-6
11	GO.0071453	cellular response to oxygen levels	3.32E-6
6	GO.0003401	axis elongation	3.35E-6
8	GO.0008630	intrinsic apoptotic signaling pathway in response to DNA damage	3.8E-6
14	GO.0001505	regulation of neurotransmitter levels	4.43E-6
12	GO.0009612	response to mechanical stimulus	4.72E-6
7	GO.2001239	regulation of extrinsic apoptotic signaling pathway in absence of ligand	4.77E-6
14	GO.0002696	positive regulation of leukocyte activation	4.88E-6
13	GO.0006469	negative regulation of protein kinase activity	5.06E-6
21	GO.0031347	regulation of defense response	5.27E-6
13	GO.0007568	aging	5.27E-6
27	GO.0046903	secretion	6.1E-6
17	GO.0061061	muscle structure development	6.46E-6
15	GO.0051098	regulation of binding	6.83E-6
18	GO.0007423	sensory organ development	7.02E-6
8	GO.2000177	regulation of neural precursor cell proliferation	7.49E-6
12	GO.0034599	cellular response to oxidative stress	7.84E-6
15	GO.1903532	positive regulation of secretion by cell	8.76E-6
16	GO.0048568	embryonic organ development	9.17E-6
14	GO.0002237	response to molecule of bacterial origin	9.3E-6
13	GO.0010948	negative regulation of cell cycle process	1.02E-5
10	GO.0010212	response to ionizing radiation	1.03E-5
16	GO.0050708	regulation of protein secretion	1.04E-5
13	GO.0070372	regulation of ERK1 and ERK2 cascade	1.05E-5
23	GO.0009894	regulation of catabolic process	1.12E-5
14	GO.0048545	response to steroid hormone	1.15E-5
5	GO.0048339	paraxial mesoderm development	1.15E-5
8	GO.0097327	response to antineoplastic agent	1.37E-5
13	GO.0071214	cellular response to abiotic stimulus	1.38E-5
4	GO.0007183	SMAD protein complex assembly	1.43E-5
8	GO.0046620	regulation of organ growth	1.47E-5
10	GO.2001242	regulation of intrinsic apoptotic signaling pathway	1.68E-5
8	GO.0009791	post-embryonic development	1.68E-5
16	GO.0010608	posttranscriptional regulation of gene expression	1.69E-5
36	GO.0003008	system process	1.91E-5
14	GO.0031346	positive regulation of cell projection organization	2.04E-5
5	GO.0060261	positive regulation of transcription initiation from RNA polymerase II promoter	2.06E-5
6	GO.0070317	negative regulation of G0 to G1 transition	2.1E-5
6	GO.0010863	positive regulation of phospholipase C activity	2.1E-5
9	GO.0002687	positive regulation of leukocyte migration	2.14E-5
7	GO.1901992	positive regulation of mitotic cell cycle phase transition	2.24E-5
6	GO.0090342	regulation of cell aging	2.67E-5
13	GO.0046677	response to antibiotic	2.92E-5
5	GO.0048103	somatic stem cell division	2.94E-5

5	GO.0030325	adrenal gland development	2.94E-5
13	GO.0001701	in utero embryonic development	3.0E-5
6	GO.1905207	regulation of cardiocyte differentiation	3.35E-5
7	GO.0030512	negative regulation of transforming growth factor beta receptor signaling pathway	3.43E-5
8	GO.0021761	limbic system development	3.51E-5
10	GO.0042594	response to starvation	3.53E-5
9	GO.0001816	cytokine production	3.69E-5
7	GO.0030279	negative regulation of ossification	3.71E-5
9	GO.0043524	negative regulation of neuron apoptotic process	3.88E-5
9	GO.0009746	response to hexose	3.88E-5
16	GO.0051493	regulation of cytoskeleton organization	4.06E-5
7	GO.0034103	regulation of tissue remodeling	4.28E-5
6	GO.0001541	ovarian follicle development	5.57E-5
6	GO.0051496	positive regulation of stress fiber assembly	6.16E-5
5	GO.0071634	regulation of transforming growth factor beta production	6.33E-5
8	GO.1900180	regulation of protein localization to nucleus	6.66E-5
20	GO.0006974	cellular response to DNA damage stimulus	6.93E-5
23	GO.0007010	cytoskeleton organization	7.06E-5
12	GO.0060249	anatomical structure homeostasis	7.18E-5
6	GO.0046626	regulation of insulin receptor signaling pathway	7.39E-5
10	GO.0061448	connective tissue development	7.64E-5
6	GO.0060688	regulation of morphogenesis of a branching structure	8.06E-5
71	GO.0051179	localization	8.15E-5
17	GO.0016032	viral process	8.64E-5
7	GO.0070661	leukocyte proliferation	8.73E-5
5	GO.0071276	cellular response to cadmium ion	9.41E-5
5	GO.0030212	hyaluronan metabolic process	9.41E-5
17	GO.0010469	regulation of signaling receptor activity	9.69E-5
9	GO.0043409	negative regulation of MAPK cascade	9.78E-5
4	GO.1905564	positive regulation of vascular endothelial cell proliferation	1.0E-4
6	GO.0072091	regulation of stem cell proliferation	1.0E-4
6	GO.0032768	regulation of monooxygenase activity	1.0E-4
6	GO.1902041	regulation of extrinsic apoptotic signaling pathway via death domain receptors	1.1E-4
7	GO.0031058	positive regulation of histone modification	1.1E-4
4	GO.2001028	positive regulation of endothelial cell chemotaxis	1.2E-4
30	GO.0065003	protein-containing complex assembly	1.2E-4
9	GO.0048167	regulation of synaptic plasticity	1.2E-4
5	GO.0043552	positive regulation of phosphatidylinositol 3-kinase activity	1.2E-4
8	GO.0030879	mammary gland development	1.2E-4
9	GO.0030324	lung development	1.2E-4
10	GO.0032872	regulation of stress-activated MAPK cascade	1.3E-4
8	GO.0032355	response to estradiol	1.3E-4
5	GO.0007616	long-term memory	1.3E-4
9	GO.1902749	regulation of cell cycle G2/M phase transition	1.4E-4
15	GO.0032990	cell part morphogenesis	1.4E-4
4	GO.1903798	regulation of production of miRNAs involved in gene silencing by miRNA	1.5E-4
18	GO.0055082	cellular chemical homeostasis	1.5E-4
9	GO.0045216	cell-cell junction organization	1.5E-4
33	GO.0044281	small molecule metabolic process	1.5E-4
5	GO.0043491	protein kinase B signaling	1.5E-4
9	GO.0042593	glucose homeostasis	1.5E-4
5	GO.0034405	response to fluid shear stress	1.5E-4
6	GO.0048015	phosphatidylinositol-mediated signaling	1.6E-4
5	GO.0033619	membrane protein proteolysis	1.6E-4
7	GO.0022612	gland morphogenesis	1.7E-4
15	GO.0006935	chemotaxis	1.9E-4
3	GO.0060011	Sertoli cell proliferation	2.1E-4
11	GO.0009895	negative regulation of catabolic process	2.2E-4
6	GO.0045670	regulation of osteoclast differentiation	2.3E-4

8	GO.0055123	digestive system development	2.4E-4
6	GO.0061333	renal tubule morphogenesis	2.5E-4
6	GO.0001837	epithelial to mesenchymal transition	2.5E-4
3	GO.0070141	response to UV-A	3.0E-4
10	GO.0045926	negative regulation of growth	3.0E-4
3	GO.0035234	ectopic germ cell programmed cell death	3.0E-4
10	GO.0003007	heart morphogenesis	3.0E-4
3	GO.0002246	wound healing involved in inflammatory response	3.0E-4
7	GO.0051781	positive regulation of cell division	3.3E-4
17	GO.0030855	epithelial cell differentiation	3.4E-4
5	GO.0033077	T cell differentiation in thymus	3.6E-4
6	GO.1900407	regulation of cellular response to oxidative stress	3.7E-4
8	GO.0007050	cell cycle arrest	3.7E-4
8	GO.0000187	activation of MAPK activity	3.7E-4
5	GO.0090199	regulation of release of cytochrome c from mitochondria	3.9E-4
5	GO.0046638	positive regulation of alpha-beta T cell differentiation	3.9E-4
6	GO.0021536	diencephalon development	4.0E-4
6	GO.0050772	positive regulation of axonogenesis	4.2E-4
6	GO.0033143	regulation of intracellular steroid hormone receptor signaling pathway	4.2E-4
9	GO.0009952	anterior/posterior pattern specification	4.2E-4
7	GO.0045667	regulation of osteoblast differentiation	4.3E-4
4	GO.0090312	positive regulation of protein deacetylation	4.5E-4
4	GO.0070723	response to cholesterol	4.5E-4
4	GO.0033688	regulation of osteoblast proliferation	4.5E-4
10	GO.0040029	regulation of gene expression, epigenetic	4.8E-4
7	GO.0001704	formation of primary germ layer	4.9E-4
4	GO.2000679	positive regulation of transcription regulatory region DNA binding	5.1E-4
4	GO.0048169	regulation of long-term neuronal synaptic plasticity	5.1E-4
10	GO.0044772	mitotic cell cycle phase transition	5.2E-4
6	GO.0042058	regulation of epidermal growth factor receptor signaling pathway	5.4E-4
5	GO.0035094	response to nicotine	5.4E-4
3	GO.0032667	regulation of interleukin-23 production	5.4E-4
6	GO.0010631	epithelial cell migration	5.4E-4
3	GO.0006268	DNA unwinding involved in DNA replication	5.4E-4
11	GO.0032102	negative regulation of response to external stimulus	5.7E-4
4	GO.2000406	positive regulation of T cell migration	5.8E-4
4	GO.1902895	positive regulation of pri-miRNA transcription by RNA polymerase II	5.8E-4
5	GO.0046632	alpha-beta T cell differentiation	5.9E-4
16	GO.0043269	regulation of ion transport	6.0E-4
6	GO.0008286	insulin receptor signaling pathway	6.0E-4
5	GO.0060071	Wnt signaling pathway, planar cell polarity pathway	6.3E-4
6	GO.0034101	erythrocyte homeostasis	6.3E-4
6	GO.0001649	osteoblast differentiation	6.3E-4
4	GO.0050927	positive regulation of positive chemotaxis	6.5E-4
4	GO.0031929	TOR signaling	6.5E-4
5	GO.0048641	regulation of skeletal muscle tissue development	6.8E-4
8	GO.0060348	bone development	6.9E-4
3	GO.0010455	positive regulation of cell fate commitment	6.9E-4
6	GO.2000106	regulation of leukocyte apoptotic process	7.0E-4
6	GO.0071158	positive regulation of cell cycle arrest	7.0E-4
4	GO.0010575	positive regulation of vascular endothelial growth factor production	7.3E-4
16	GO.0051129	negative regulation of cellular component organization	7.4E-4
9	GO.0021700	developmental maturation	7.4E-4
16	GO.0007276	gamete generation	7.4E-4
5	GO.0061515	myeloid cell development	7.8E-4
10	GO.0009615	response to virus	7.9E-4
4	GO.0035196	production of miRNAs involved in gene silencing by miRNA	8.1E-4

3	GO.1900122	positive regulation of receptor binding	8.6E-4
3	GO.0060068	vagina development	8.6E-4
3	GO.0051574	positive regulation of histone H3-K9 methylation	8.6E-4
3	GO.0048143	astrocyte activation	8.6E-4
3	GO.0045792	negative regulation of cell size	8.6E-4
6	GO.0045639	positive regulation of myeloid cell differentiation	8.6E-4
6	GO.0009408	response to heat	8.6E-4
14	GO.0051259	protein complex oligomerization	8.9E-4
4	GO.0048147	negative regulation of fibroblast proliferation	8.9E-4
4	GO.0040036	regulation of fibroblast growth factor receptor signaling pathway	8.9E-4
13	GO.0051051	negative regulation of transport	9.0E-4
7	GO.0046887	positive regulation of hormone secretion	9.0E-4
22	GO.1901135	carbohydrate derivative metabolic process	9.3E-4
8	GO.0071216	cellular response to biotic stimulus	9.5E-4
4	GO.0071542	dopaminergic neuron differentiation	9.9E-4
4	GO.0033280	response to vitamin D	9.9E-4
6	GO.0060070	canonical Wnt signaling pathway	0.001
3	GO.2000615	regulation of histone H3-K9 acetylation	0.0011
3	GO.0014745	negative regulation of muscle adaptation	0.0011
4	GO.1905332	positive regulation of morphogenesis of an epithelium	0.0012
4	GO.0090398	cellular senescence	0.0012
5	GO.0061098	positive regulation of protein tyrosine kinase activity	0.0012
5	GO.0060135	maternal process involved in female pregnancy	0.0012
4	GO.0051150	regulation of smooth muscle cell differentiation	0.0012
4	GO.0051145	smooth muscle cell differentiation	0.0012
4	GO.0043537	negative regulation of blood vessel endothelial cell migration	0.0012
5	GO.0019229	regulation of vasoconstriction	0.0012
10	GO.0010506	regulation of autophagy	0.0012
5	GO.0006940	regulation of smooth muscle contraction	0.0012
4	GO.0048873	homeostasis of number of cells within a tissue	0.0013
3	GO.0043149	stress fiber assembly	0.0013
4	GO.0043029	T cell homeostasis	0.0013
5	GO.0042698	ovulation cycle	0.0013
4	GO.0010165	response to X-ray	0.0013
8	GO.0002699	positive regulation of immune effector process	0.0013
5	GO.0061035	regulation of cartilage development	0.0014
5	GO.0021872	forebrain generation of neurons	0.0014
4	GO.1900271	regulation of long-term synaptic potentiation	0.0015
4	GO.0097421	liver regeneration	0.0015
3	GO.0032740	positive regulation of interleukin-17 production	0.0015
10	GO.0030198	extracellular matrix organization	0.0015
6	GO.0006282	regulation of DNA repair	0.0015
10	GO.0001525	angiogenesis	0.0015
6	GO.0043200	response to amino acid	0.0016
4	GO.0030501	positive regulation of bone mineralization	0.0016
13	GO.0006954	inflammatory response	0.0016
6	GO.0002573	myeloid leukocyte differentiation	0.0016
5	GO.2000736	regulation of stem cell differentiation	0.0017
5	GO.0051785	positive regulation of nuclear division	0.0017
5	GO.0043618	regulation of transcription from RNA polymerase II promoter in response to stress	0.0017
4	GO.0035924	cellular response to vascular endothelial growth factor stimulus	0.0017
4	GO.0032885	regulation of polysaccharide biosynthetic process	0.0017
5	GO.1901224	positive regulation of NIK/NF-kappaB signaling	0.0019
2	GO.0032916	positive regulation of transforming growth factor beta3 production	0.002
3	GO.2001267	regulation of cysteine-type endopeptidase activity involved in apoptotic signaling pathway	0.0021
7	GO.0019318	hexose metabolic process	0.0021
8	GO.0048705	skeletal system morphogenesis	0.0022

7	GO.0030178	negative regulation of Wnt signaling pathway	0.0022
7	GO.0007265	Ras protein signal transduction	0.0022
6	GO.0032273	positive regulation of protein polymerization	0.0023
5	GO.0031016	pancreas development	0.0023
6	GO.0006941	striated muscle contraction	0.0023
3	GO.0051481	negative regulation of cytosolic calcium ion concentration	0.0024
4	GO.0051402	neuron apoptotic process	0.0024
5	GO.0043627	response to estrogen	0.0025
7	GO.0031589	cell-substrate adhesion	0.0027
11	GO.0044282	small molecule catabolic process	0.0028
6	GO.0050864	regulation of B cell activation	0.0029
6	GO.0001764	neuron migration	0.0029
8	GO.0034612	response to tumor necrosis factor	0.003
4	GO.1990090	cellular response to nerve growth factor stimulus	0.0031
3	GO.0060749	mammary gland alveolus development	0.0031
4	GO.0060425	lung morphogenesis	0.0031
4	GO.0048662	negative regulation of smooth muscle cell proliferation	0.0031
4	GO.0035272	exocrine system development	0.0031
2	GO.0032227	negative regulation of synaptic transmission, dopaminergic	0.0031
6	GO.0030168	platelet activation	0.0031
3	GO.0010592	positive regulation of lamellipodium assembly	0.0031
5	GO.0055007	cardiac muscle cell differentiation	0.0032
4	GO.0045843	negative regulation of striated muscle tissue development	0.0034
3	GO.2000178	negative regulation of neural precursor cell proliferation	0.0035
6	GO.0043280	positive regulation of cysteine-type endopeptidase activity involved in apoptotic process	0.0035
7	GO.0030522	intracellular receptor signaling pathway	0.0037
7	GO.0070507	regulation of microtubule cytoskeleton organization	0.0042
7	GO.0008217	regulation of blood pressure	0.0042
2	GO.2000741	positive regulation of mesenchymal stem cell differentiation	0.0043
3	GO.2000637	positive regulation of gene silencing by miRNA	0.0043
4	GO.1904705	regulation of vascular smooth muscle cell proliferation	0.0043
6	GO.0062013	positive regulation of small molecule metabolic process	0.0043
2	GO.0060665	regulation of branching involved in salivary gland morphogenesis by mesenchymal-epithelial signaling	0.0043
3	GO.0060444	branching involved in mammary gland duct morphogenesis	0.0043
2	GO.0021764	amygdala development	0.0043
5	GO.0019217	regulation of fatty acid metabolic process	0.0043
2	GO.0014042	positive regulation of neuron maturation	0.0043
4	GO.0002762	negative regulation of myeloid leukocyte differentiation	0.0043
17	GO.0007155	cell adhesion	0.0044
6	GO.0048863	stem cell differentiation	0.0045
4	GO.2000378	negative regulation of reactive oxygen species metabolic process	0.0046
4	GO.0051148	negative regulation of muscle cell differentiation	0.0046
3	GO.0010288	response to lead ion	0.0048
6	GO.0061013	regulation of mRNA catabolic process	0.005
6	GO.0009306	protein secretion	0.005
9	GO.0010721	negative regulation of cell development	0.0051
4	GO.0007173	epidermal growth factor receptor signaling pathway	0.0051
5	GO.1990830	cellular response to leukemia inhibitory factor	0.0053
3	GO.1902667	regulation of axon guidance	0.0053
3	GO.0071379	cellular response to prostaglandin stimulus	0.0053
3	GO.0062009	secondary palate development	0.0053
5	GO.0044070	regulation of anion transport	0.0053
4	GO.0032655	regulation of interleukin-12 production	0.0054
10	GO.0031349	positive regulation of defense response	0.0055
2	GO.1903223	positive regulation of oxidative stress-induced neuron death	0.0056
13	GO.0044283	small molecule biosynthetic process	0.0056
6	GO.0031098	stress-activated protein kinase signaling cascade	0.0056
2	GO.0018401	peptidyl-proline hydroxylation to 4-hydroxy-L-proline	0.0056
2	GO.0002551	mast cell chemotaxis	0.0056

5	GO.0062014	negative regulation of small molecule metabolic process	0.0057
5	GO.0042552	myelination	0.0057
8	GO.0007162	negative regulation of cell adhesion	0.0057
8	GO.0001818	negative regulation of cytokine production	0.0057
5	GO.1902850	microtubule cytoskeleton organization involved in mitosis	0.006
8	GO.0090596	sensory organ morphogenesis	0.006
5	GO.0072527	pyrimidine-containing compound metabolic process	0.006
5	GO.0048771	tissue remodeling	0.006
6	GO.0051092	positive regulation of NF-kappaB transcription factor activity	0.0064
3	GO.0021591	ventricular system development	0.0064
7	GO.0097237	cellular response to toxic substance	0.0065
4	GO.0031532	actin cytoskeleton reorganization	0.0067
5	GO.0014066	regulation of phosphatidylinositol 3-kinase signaling	0.0067
3	GO.0070306	lens fiber cell differentiation	0.007
3	GO.0060251	regulation of glial cell proliferation	0.007
2	GO.0060527	prostate epithelial cord arborization involved in prostate glandular acinus morphogenesis	0.0071
14	GO.0055086	nucleobase-containing small molecule metabolic process	0.0071
2	GO.0042713	sperm ejaculation	0.0071
2	GO.0031536	positive regulation of exit from mitosis	0.0071
2	GO.0030579	ubiquitin-dependent SMAD protein catabolic process	0.0071
6	GO.0070555	response to interleukin-1	0.0072
10	GO.0097435	supramolecular fiber organization	0.0073
4	GO.0045600	positive regulation of fat cell differentiation	0.0073
5	GO.0045444	fat cell differentiation	0.0074
3	GO.0031128	developmental induction	0.0074
9	GO.0022412	cellular process involved in reproduction in multicellular organism	0.0074
3	GO.0001702	gastrulation with mouth forming second	0.0074
5	GO.2000181	negative regulation of blood vessel morphogenesis	0.0076
4	GO.0042531	positive regulation of tyrosine phosphorylation of STAT protein	0.0077
8	GO.0009410	response to xenobiotic stimulus	0.0079
5	GO.0072593	reactive oxygen species metabolic process	0.0081
3	GO.0033032	regulation of myeloid cell apoptotic process	0.0081
4	GO.0021885	forebrain cell migration	0.0081
5	GO.0021510	spinal cord development	0.0081
4	GO.0014910	regulation of smooth muscle cell migration	0.0081
2	GO.2000138	positive regulation of cell proliferation involved in heart morphogenesis	0.0087
2	GO.0090270	regulation of fibroblast growth factor production	0.0087
2	GO.0042661	regulation of mesodermal cell fate specification	0.0087
2	GO.0031077	post-embryonic camera-type eye development	0.0087
13	GO.0007017	microtubule-based process	0.0087
6	GO.0051701	interaction with host	0.0092
6	GO.0006338	chromatin remodeling	0.0092
3	GO.1900181	negative regulation of protein localization to nucleus	0.0093
5	GO.0071773	cellular response to BMP stimulus	0.0093
3	GO.0030947	regulation of vascular endothelial growth factor receptor signaling pathway	0.0093
6	GO.0002695	negative regulation of leukocyte activation	0.0093
3	GO.0001662	behavioral fear response	0.0093
4	GO.0071333	cellular response to glucose stimulus	0.0096
4	GO.0051926	negative regulation of calcium ion transport	0.0096
7	GO.0043393	regulation of protein binding	0.0096
4	GO.0001570	vasculogenesis	0.0096
5	GO.0021987	cerebral cortex development	0.0098
17	GO.0002252	immune effector process	0.0098

Table S4. KEGG pathways enrichment results for terms with FDR *P*<0.01

Number of Genes	Term name	Description	FDR
46	hsa05200	Pathways in cancer	5.42E-30
30	hsa05206	MicroRNAs in cancer	2.45E-28
29	hsa05205	Proteoglycans in cancer	3.71E-24
34	hsa04151	PI3K-Akt signaling pathway	3.18E-23
20	hsa05212	Pancreatic cancer	4.66E-21
23	hsa05224	Breast cancer	9.96E-20
22	hsa04218	Cellular senescence	4.46E-18
18	hsa05215	Prostate cancer	1.09E-16
18	hsa04933	AGE-RAGE signaling pathway in diabetic complications	1.18E-16
24	hsa05166	HTLV-I infection	2.15E-16
25	hsa04010	MAPK signaling pathway	4.93E-16
16	hsa01521	EGFR tyrosine kinase inhibitor resistance	1.32E-15
18	hsa04068	FoxO signaling pathway	6.47E-15
17	hsa04550	Signaling pathways regulating pluripotency of stem cells	2.26E-13
16	hsa05418	Fluid shear stress and atherosclerosis	1.61E-12
15	hsa04110	Cell cycle	7.32E-12
13	hsa05222	Small cell lung cancer	4.61E-11
15	hsa04150	mTOR signaling pathway	7.55E-11
13	hsa04066	HIF-1 signaling pathway	8.58E-11
16	hsa05203	Viral carcinogenesis	1.11E-10
15	hsa04630	Jak-STAT signaling pathway	1.91E-10
14	hsa04910	Insulin signaling pathway	2.32E-10
16	hsa04510	Focal adhesion	2.83E-10
11	hsa05230	Central carbon metabolism in cancer	2.85E-10
12	hsa04211	Longevity regulating pathway	3.33E-10
14	hsa04310	Wnt signaling pathway	4.37E-10
13	hsa04722	Neurotrophin signaling pathway	4.46E-10
13	hsa04926	Relaxin signaling pathway	1.6E-9
9	hsa05219	Bladder cancer	1.93E-9
13	hsa05162	Measles	1.98E-9
10	hsa05221	Acute myeloid leukemia	4.61E-9
10	hsa05211	Renal cell carcinoma	5.86E-9
10	hsa04917	Prolactin signaling pathway	6.53E-9
10	hsa04520	Adherens junction	7.92E-9
12	hsa04371	Apelin signaling pathway	1.88E-8
9	hsa05213	Endometrial cancer	2.36E-8
13	hsa05152	Tuberculosis	2.9E-8
11	hsa04919	Thyroid hormone signaling pathway	4.43E-8
11	hsa04380	Osteoclast differentiation	8.98E-8
10	hsa04668	TNF signaling pathway	2.7E-7
9	hsa04350	TGF-beta signaling pathway	3.54E-7
11	hsa04934	Cushing's syndrome	6.23E-7
10	hsa05160	Hepatitis C	1.35E-6
9	hsa04660	T cell receptor signaling pathway	1.35E-6
10	hsa04915	Estrogen signaling pathway	1.5E-6
9	hsa05142	Chagas disease (American trypanosomiasis)	1.51E-6
7	hsa05321	Inflammatory bowel disease (IBD)	6.87E-6
9	hsa04140	Autophagy - animal	7.61E-6
10	hsa05202	Transcriptional misregulation in cancer	1.08E-5
7	hsa04920	Adipocytokine signaling pathway	1.25E-5
8	hsa04922	Glucagon signaling pathway	1.29E-5
9	hsa04932	Non-alcoholic fatty liver disease (NAFLD)	2.63E-5
9	hsa04022	cGMP-PKG signaling pathway	4.41E-5
11	hsa04060	Cytokine-cytokine receptor interaction	7.03E-5
6	hsa05217	Basal cell carcinoma	7.8E-5
7	hsa04916	Melanogenesis	9.3E-5

6	hsa05120	Epithelial cell signaling in Helicobacter pylori infection	9.72E-5
6	hsa05140	Leishmaniasis	1.3E-4
5	hsa04930	Type II diabetes mellitus	1.9E-4
5	hsa05014	Amyotrophic lateral sclerosis (ALS)	2.7E-4
7	hsa04921	Oxytocin signaling pathway	9.6E-4
4	hsa05216	Thyroid cancer	9.9E-4
4	hsa04973	Carbohydrate digestion and absorption	0.0015
4	hsa04340	Hedgehog signaling pathway	0.0021
4	hsa04330	Notch signaling pathway	0.0024
5	hsa04666	Fc gamma R-mediated phagocytosis	0.0029
4	hsa04923	Regulation of lipolysis in adipocytes	0.0033
5	hsa04657	IL-17 signaling pathway	0.0033
6	hsa04261	Adrenergic signaling in cardiomyocytes	0.0035
7	hsa04810	Regulation of actin cytoskeleton	0.0052
5	hsa04726	Serotonergic synapse	0.0072
5	hsa04670	Leukocyte transendothelial migration	0.0072
6	hsa04530	Tight junction	0.0078
7	hsa04714	Thermogenesis	0.0086
4	hsa05133	Pertussis	0.0093
3	hsa04960	Aldosterone-regulated sodium reabsorption	0.0098

Table S5. Reactome pathways enrichment results for terms with FDR *P*<0.01

Number of Genes	Term name	Description	FDR
31	HSA-9006934	Signaling by Receptor Tyrosine Kinases	2.06E-16
19	HSA-6785807	Interleukin-4 and Interleukin-13 signaling	2.91E-16
27	HSA-5663202	Diseases of signal transduction	6.66E-15
43	HSA-212436	Generic Transcription Pathway	1.45E-14
13	HSA-2219528	PI3K/AKT Signaling in Cancer	1.87E-10
10	HSA-8848021	Signaling by PTK6	7.28E-9
46	HSA-168256	Immune System	8.36E-9
12	HSA-194138	Signaling by VEGF	1.76E-8
12	HSA-453279	Mitotic G1-G1/S phases	4.76E-7
13	HSA-157118	Signaling by NOTCH	1.24E-6
10	HSA-8878166	Transcriptional regulation by RUNX2	4.49E-6
16	HSA-3700989	Transcriptional Regulation by TP53	5.16E-6
27	HSA-1266738	Developmental Biology	8.09E-6
6	HSA-114452	Activation of BH3-only proteins	1.39E-5
5	HSA-8934593	Regulation of RUNX1 Expression and Activity	2.78E-5
13	HSA-5683057	MAPK family signaling cascades	2.78E-5
6	HSA-6804757	Regulation of TP53 Degradation	2.87E-5
19	HSA-109582	Hemostasis	3.2E-5
8	HSA-8878159	Transcriptional regulation by RUNX3	4.07E-5
10	HSA-2559583	Cellular Senescence	4.08E-5
13	HSA-195721	Signaling by WNT	4.31E-5
6	HSA-1433557	Signaling by SCF-KIT	4.52E-5
6	HSA-1236394	Signaling by ERBB4	5.71E-5
4	HSA-8866910	TFAP2 (AP-2) family regulates transcription of growth factors and their receptors	9.88E-5
4	HSA-877312	Regulation of IFNG signaling	1.3E-4
6	HSA-8986944	Transcriptional Regulation by MECP2	2.2E-4
4	HSA-3772470	Negative regulation of TCF-dependent signaling by WNT ligand antagonists	2.2E-4
3	HSA-211163	AKT-mediated inactivation of FOXO1A	2.5E-4
7	HSA-166520	Signaling by NTRKs	3.5E-4
3	HSA-8849470	PTK6 Regulates Cell Cycle	5.2E-4
5	HSA-177929	Signaling by EGFR	5.2E-4
5	HSA-1227986	Signaling by ERBB2	7.7E-4
3	HSA-8951936	RUNX3 regulates p14-ARF	0.0011
4	HSA-1538133	G0 and Early G1	0.0012
5	HSA-168643	Nucleotide-binding domain, leucine rich repeat containing receptor (NLR) signaling pathways	0.0013
10	HSA-5688426	Deubiquitination	0.0017
3	HSA-9013700	NOTCH4 Activation and Transmission of Signal to the Nucleus	0.0018
7	HSA-73887	Death Receptor Signalling	0.002
3	HSA-75035	Chk1/Chk2(Cds1) mediated inactivation of Cyclin B:Cdk1 complex	0.0024
3	HSA-8849471	PTK6 Regulates RHO GTPases, RAS GTPase and MAP kinases	0.0029
3	HSA-6804116	TP53 Regulates Transcription of Genes Involved in G1 Cell Cycle Arrest	0.0029
4	HSA-5689896	Ovarian tumor domain proteases	0.0032
3	HSA-2173791	TGF-beta receptor signaling in EMT (epithelial to mesenchymal transition)	0.0038
3	HSA-881907	Gastrin-CREB signalling pathway via PKC and MAPK	0.0043
3	HSA-450604	KSRP (KHSRP) binds and destabilizes mRNA	0.0043
3	HSA-445144	Signal transduction by L1	0.0061
5	HSA-381340	Transcriptional regulation of white adipocyte differentiation	0.0061
2	HSA-9032759	NTRK2 activates RAC1	0.0066
2	HSA-209563	Axonal growth stimulation	0.0066
4	HSA-3928665	EPH-ephrin mediated repulsion of cells	0.0069
5	HSA-6804756	Regulation of TP53 Activity through Phosphorylation	0.0084
